# Superconducting YBCO Foams as Trapped Field Magnets

**DOI:** 10.3390/ma12060853

**Published:** 2019-03-13

**Authors:** Michael R. Koblischka, Sugali Pavan Kumar Naik, Anjela Koblischka-Veneva, Masato Murakami, Denis Gokhfeld, Eddula Sudhakar Reddy, Georg J. Schmitz

**Affiliations:** 1 Superconducting Materials Laboratory, Department of Materials Science and Engineering, Shibaura Institute of Technology, Tokyo 135-8548, Japan; spavankumarnaik@yahoo.in (S.P.K.N.); anjela@shibaura-it.ac.jp (A.K.-V.); masatomu@shibaura-it.ac.jp (M.M.); 2 Kirensky Institute of Physics, Federal Research Center KSC SB RAS, Krasnoyarsk 660036, Russia; gokhfeld@yandex.ru; 3 ACCESS, Intzestrasse 5, 52072 Aachen, Germany; sudhakar_reddy@raychemrpg.com (E.S.R.); g.schmitz@access-technology.de (G.J.S.)

**Keywords:** High-*T_c_* superconductors, YBCO, foam, trapped fields, current flow

## Abstract

Superconducting foams of YBa_2_Cu_3_O_*y*_ (YBCO) are proposed as trapped field magnets or supermagnets. The foams with an open-porous structure are light-weight, mechanically strong and can be prepared in large sample sizes. The trapped field distributions were measured using a scanning Hall probe on various sides of an YBCO foam sample after field-cooling in a magnetic field of 0.5 T produced by a square Nd-Fe-B permanent magnet. The maximum trapped field (TF) measured is about 400 G (77 K) at the bottom of the sample. Several details of the TF distribution, the current flow and possible applicatons of such superconducting foam samples in space applications, e.g., as active elements in flux-pinning docking interfaces (FPDI) or as portable strong magnets to collect debris in space, are outlined.

## 1. Introduction

Today, high-$T_{c}$ superconductor samples are mainly fabricated in three different shapes: wires/tapes, thin films and bulk materials [1,2,3]. The wires and tapes serve the purpose of energy transport or building of large electromagnets, the thin films cover the needs of superconducting electronics and sensors, and the bulk materials are employed for levitation, in electric motors and generators or as trapped field magnets (superconducting permanent magnets), which can be much stronger as any permanent magnet material. For most applications operating at liquid nitrogen temperature (77 K), YBa_2_Cu_3_O_*y*_ (YBCO) is the material of choice due to its superior properties at elevated temperatures [4].

For building superconducting trapped field (TF) magnets or “supermagnets”, a large sample size is required as the maximum trapped field depends on the sample size in contrast to permanent magnets [5,6]. However, obtaining large sample sizes is a difficult task as a good texture is essential to enable the flow of strong supercurrents. The necessary oxygenation step when preparing YBCO leads to a phase transformation from a tetragonal phase (YBa_2_Cu_3_O_6…6.5_) to an orthorhombic phase (YBa_2_Cu_3_O_6.6…7_). In this step, internal cracking may occur which limits the possible sample size [7]. Furthermore, the oxygenation time required to fully oxygenate a large, bulk sample increases tremendously; so necessary oxygenation times of 14 days and more are quite common. Therefore, superconducting samples prepared in this way are costly, and difficult to be handled. Furthermore, magnetostriction plays an important role when trapping large magnetic fields [6]. Due to the forces involved, the superconductor sample may break up, and the aforementioned internal cracks strongly contribute to this. Therefore, a reinforcement by steel rings and additional polymer impregnation is required to withstand the forces [8,9,10]. In this way, a record trapped field of 17.6 T at 26 K was reached in the literature, measured in between a stack of two melt-textured superconductor samples with 25 mm diameter.

An alternative preparation route for bulk superconductors would be very interesting to solve the problems mentioned. A possible way out was attempted several years ago with the preparation of superconducting foams. Polymer, metallic or ceramic foams as nature-inspired bionic materials mimicing the construction elements of biologic load-bearing structures like wood or bones are nowadays used in many places in industry for various tasks, e.g., as energy absorbers in aeronautics or in car industry, as light-weight structural damping material, as filter materials, and others. These uses were recently reviewed in References [11,12,13]. The open-porous structure of foams enables the addition of metallic layers by, e.g., electrodeposition to further improve the mechanical strength [14], and the pores may be filled up with resins. Such treatment could be very important for future TF magnets based on superconducting foams. Furthermore, the foam-type material enables an easy shaping of the samples and upscaling of the sample size [15].

The YBCO foams were originally intended to be used as fault current limiters [16,17], as the open porous structure of the foam materials enables a very effective cooling process. Liquid nitrogen or any other coolant in form of liquid or gas can directly be sent through the sample. Therefore, possible hotspots in the material can be effectively cooled down again [11]. This very effective cooling can be easily demonstrated when cooling a foam sample in liquid nitrogen to perform, e.g., levitation experiments as shown in the Supplementary Materials. The superconducting foams can be applied as trapped field magnets, as the easy upscaling enables to create foam samples of larger dimensions as the conventional bulk samples, avoiding the extremely long oxygenation times required. Furthermore, the foams have much less weight as compared to the bulks, and less material is required reducing the costs of a sample further. These advantages make the superconducting foams ideal candidates for applications in space. This may comprise the flux-pinning docking interfaces (FPDI) discussed in the literature [18,19] and portable high-magnetic field units which can be mounted in satellites to magnetically collect waste debris [20,21,22].

In this contribution, we present the results of trapped field measurements at 77 K on a superconducting large YBCO foam piece for the first time, as in a previous paper [23] only small foam pieces were measured. Here, we further investigate the effect of magnetizing the foam on different sample sides to gain information on the texture properties of the bulk sample. Furthermore, the microstructure of the foam struts is investigated using the electron backscatter diffraction (EBSD) technique. The results obtained are intended to set a benchmark for the next generation of foam samples to be prepared. Several possible applications of such foam samples are discussed as well.

## 2. Experimental Procedures

### 2.1. Sample Preparation

**Figure 1:**
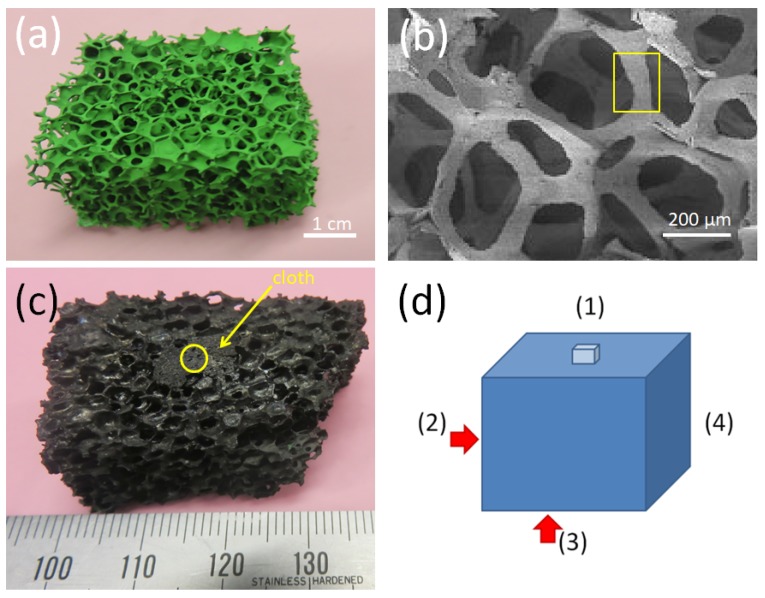
(**a**) Image of the preform stage: A green 211 foam. (**b**) SEM image of the 3D structure of the 211 foam. The yellow rectangle marks the central piece of a strut as analyzed by magnetic measurements and for the microstructure investigation. (**c**) presents the fully reacted, superconducting foam. On the top Side (1), the remains of the seed crystal (yellow circle) and a YBCO cloth (yellow arrow) are still visible. (**d**) gives a schematic drawing of the foam sample and the nomenclature of the sample sides.

The foam samples were prepared at RWTH Aachen, Germany. The preparation procedure was described in detail in References [16,17]. Starting from a polyurethane foam, a preform Y_2_BaCuO_5_ (211) foam was created by impregnating the foam piece with a 211 slurry, formed by mixing commercially available 211 powder (1–5 μm grain size) in a water-based solution with 5 wt.% polyvinylalcohol as binder. A thermal process follows such that (i) the organic components (polyvinylalcohol and polyurethane) are burnt off by slow heating at 50 K/h to 600 °C and dwelling for 6 h, and the resulting 211 ceramic is densified by further heating at 150 K/h to 1100 °C. The result is a stable 211 structure being presented in Figure 1a. Figure 1b gives a SEM image of the 3D-structure of the 211 foam being a copy of the structure of the original polyurethane foam. The yellow box marks a foam strut. Struts like this were broken off the sample for measurements of magnetization loops and the superconducting transition temperature. The next step of preparation is a transformation of the 211 foam into a superconducting YBCO foam. Here it is important to point out that crystallographic orientation was introduced to the foam by using the infiltration growth process together with a seed crystal (here, a small NdBa_2_Cu_3_O_*x*_ crystal was employed) on top of the foam structure. The entire assembly is heated up above the peritectic temperature (1010 °C). The YBa_2_Cu_3_O_7_ phase decomposes into solid Y_2_BaCuO_5_ and liquid phase, which infiltrates into the Y_2_BaCuO_5_ skeleton by capillarity. The infiltrated foam is then slowly cooled down (cooling rate 0.3 K/h) through the peritectic temperature with the seed crystal on top. To prevent the seed crystal from topping off, an YBCO cloth was used. Such superconducting cloths were fabricated using a commercial Y_2_O_3_ cloth as a base [24]. Finally, the foam sample used in the TF measurements is presented in Figure 1c. The yellow circle marks the position of the original seed crystal (the remnants of which can still be seen on top). Furthermore, the arrow points to the YBCO cloth placed below the seed crystal. This treatment introduced indeed a texture as evidenced by X-ray and neutron diffraction [25]. Figure 1d presents a schematic drawing of the sample with the nomenclature of the sample sides: The side with the former seed crystal is labelled Side (1), and then we proceed by turning the sample clockwise by 90°, so that Side (2) faces to the top; Side (3) corresponds to the bottom side (where still some traces of the former liquid source can be found), and Sides (2) and (4) are the short sides of the foam sample. This way of measurement was chosen to see which of the sample sides yields the best TF properties, whereas former TF experiments on foam samples only considered the top Side (1), in analogy to the conventional bulk samples [23].

The superconducting transition temperature, $T_{c}$, of the foam samples as determined by magnetic measurements is ∼91 K like in the bulk YBCO samples.

### 2.2. Magnetic Measurements

Magnetization loops (MHLs) were recorded using SQUID magnetometry (Quantum Design MPMS3 magnetometer with ±7 T maximum field) on individual foam strut pieces. The magnetic field was applied perpendicular to the surface of the foam strut. The field sweep rate was 0.36 T/min. The critical current densities were obtained from the magnetization data using the critical state model [26].

**Figure 2:**
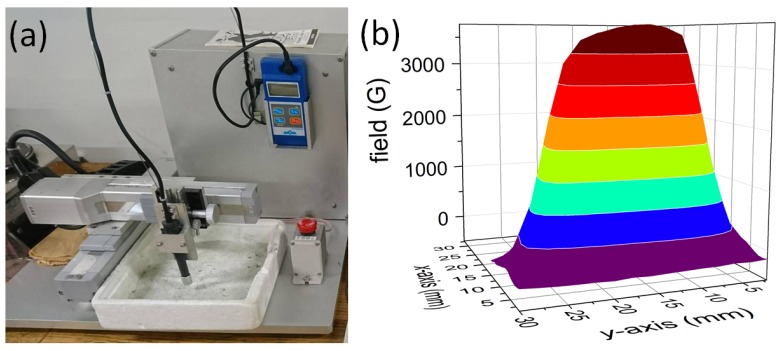
(**a**) Image of the home-built TF measurement setup; (**b**) Field distribution above the square-shaped permanent magnet (30 × 30 × 25 mm^3^) applied to magnetize the foam sample.

Trapped field (TF) measurement of the large foam piece (5 × 2 × 2 cm^3^) were carried out using a homemade setup with a scanning Hall probe operating at 77 K. The foam sample was field cooled (FC) in a field of a commercial, Nd-Fe-B permanent magnet (dimensions of 30 × 30 × 25 mm^3^, 0.5 T surface field, remanent magnetization 1.3 T) in a liquid nitrogen bath. The TF values were recorded using a Hall probe (size of the active element 100 × 100 μm^2^, sensitivity >5 mV/T) connected to a Gauss meter (MAGNA model MG-601). The step size in x,y-directions is 1 mm. A view of this setup is presented in Figure 2a, together with the field distribution produced by the permanent magnet (b). For each measurement in this paper, the permanent magnet was placed above the side analyzed. TF flux density profiles were recorded after 15 min waiting time at 1.5–2 mm height above the foam surface as the surface of the foam is not fully flat, and no surface treatment was carried out. Therefore, the obtained TF values are slightly smaller than that of other melt-textured bulk samples, which are measured at 1 mm distance. Critical current densities were obtained from the TF data with an approximation of Chen’s formula [27].

The microstructure investigations were performed using scanning electron microscopy (SEM), atomic force microscopy (AFM), and electron backscatter diffraction (EBSD). More details about these measurements can be found in previous publications [28,29].

## 3. Results and Discussion

### 3.1. Trapped Fields

**Figure 3:**
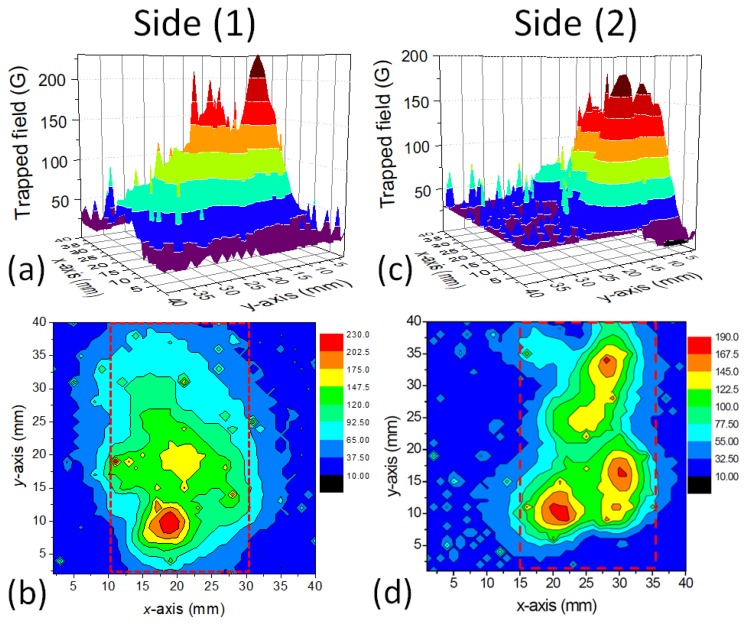
Trapped field measurements on a foam sample. Distribution of $B_{z}$ measured by scanning Hall probe on different sample sides of the foam as surface plots (**a**,**c**) and contour plots (**b**,**d**). (**a**,**b**) present the data of sample Side (1), (**c**,**d**) sample Side (2). The sample position is marked in the contour plots by a dotted red line. The permanent magnet in the FC cooling process was placed on each side in a separate measurement run.

**Figure 4:**
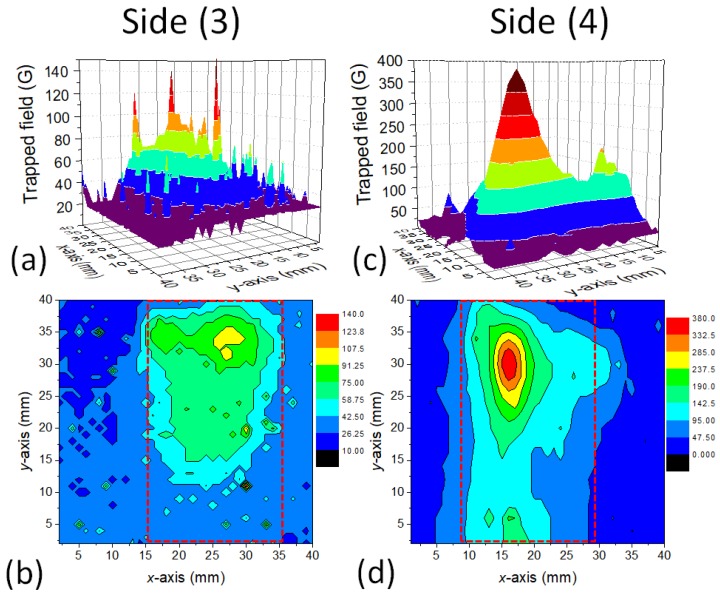
Trapped field measurements on a foam sample. Distribution of $B_{z}$ measured by scanning Hall probe on the sample Sides (3) and (4) of the foam as surface plots (**a**,**c**) and contour plots (**b**,**d**). (**a**,**b**) present the data of sample Side (3), (**c**,**d**) sample Side (4). The sample position is marked in the contour plots by a dotted red line.

Figure 3 and Figure 4 show the results of a trapped field experiment on the YBCO foam sample. Field-cooling (FC) was performed in the field of a square Nd–Fe–B magnet with a surface field of 0.5 T. For each measurement, the magnet was placed above the investigated sample side, and field-cooling was performed in this way.

Figure 3 gives the measured trapped field distributions as 3D surface plots (a,c) as well as contour plots (b,d) for the foam sample surface (Side (1)) and for the Side (2). Figure 4 shows the measurement results for Sides (3) and (4). On all sample sides, we find a large trapped field peak, and characteristically, also the presence of several sharp, small peaks. The foam Sides (1) and (2) exhibit several smaller peaks with only a small value of $B_{\mathrm{trap}}$, whereas the Sides (3) and (4) show broad TF peaks. On Side (4), the maximum trapped field of 400 G is recorded. This is a surprising result, as we would have expected the maximum trapped field either on Side (1) or on Side (3), as position-dependent measurements of the properties of foam struts had shown increasing $j_{c}$ towards the bottom of the foam sample [30]. Remarkably, there are many of the small, sharp peaks especially on Side (3), the bottom side of the foam sample.

The TF measurements clearly demonstrate the presence of two types of currents in the foam sample—one current density, which is running through the entire sample perimeter, resulting in the large, cone-shaped maxima with $B_{\mathrm{trap},\max}$, and one locally distributed current density being responsible for the small, sharp peaks. Both currents are percolative, truely 3D currents flowing through the foam struts, and are not confined to planes in the sample. Repeating the scan of $B_{z}$ reproduces most of these peaks, but the height of the peaks may vary. We ascribe these peaks to local current loops in the sample formed around local defects (e.g., large pores, pore clusters or non-superconducting struts), and are compressed due to the internal field, $B_{i}$, induced by circulating currents when removing the external field. Similar observations were made already in Reference [23], where only the surface (Side (1)) close to the seed crystal was measured. Here we have to note that the Hall probes measure only the *z*-component of the internal field $B_{i}$ generated by the flowing currents, and not the currents themselves. This implies that current paths along the *z*-axis without a *z*-component of the generated magnetic field will not be detected. Only the *(x,y)*-projection of the real circulating 3D currents will be measured, and, the various contributions to it are weighted relative to their distance to the scanning Hall probes. This point is essential to keep in mind when comparing the local current densities (e.g., measured on the foam struts) with the overall current density of the entire foam sample.

The TF measurements on the four different sample sides give important input for the optimization of the foams in future experiments. According to Reference [31], where the relation between sample geometry and TF values of melt-textured bulks were investigated, all parts of the sample contribute to the recorded TF value. In this paper, it was shown that the removal of the bottom layer of a bulk sample influences the overall measured trapped field. This is certainly even more true for the present foam sample with its 3D structure.

The present trapped field values recorded in our present experiments are not spectacular, as the values are still much smaller than those obtained on YBCO or GdBCO bulk samples at 77 K. The current records are about 800 mT (SmBCO) and 1.1 T (GdBCO) when cooling the sample to 77 K in 1.3/1.6 T applied magnetic field using an electromagnet [32,33]. In case of samples magnetized by a permanent magnet, the TF fields of bulks range between 150 [34] and 420 mT [35], depending on the sample size and the chemical additions to create additional flux pinning sites. The differences in the TF values obtained are due to the differences in the magnetization process: An electromagnet produces a homogeneous field between the pole pieces, whereas a permanent magnet has an inhomogeneous field as shown in Figure 2. The magnetic field of a permanent magnet decreases with an increase in the distance from its surface, and, the field possesses all (three) components of the magnetic field. Due to this, the flux trapping behavior is different. Therefore, the present foam data should be compared especially to those of Reference [34], which describe a similar measurement situation. Moreover, the samples are stemming from the same period discussing the first TF data on IG-grown YBCO bulks.

Our present results were influenced by several factors:

- The large sample size of the foam piece employed in the present paper did not allow the use of an electromagnet due to the limited space between the pole shoes.
- The present foam sample was pure YBCO with no extra flux-pinning sites.
- The current shape of the foams was never optimized to reach large trapped fields, nor was the texture of the sample fully optimized.

X-ray and neutron diffraction [25] have shown that a dominating orientation like the conventional bulk samples in *c*-direction is reached, but magnetization measurements even on single struts still reveal signs of granularity [36]. The first TF experiments on foams carried out in 2003 [23] had shown trapped fields of ∼400 G, when field-cooling the foam sample in a field of 600 mT on Side (1) only. In these experiments, it was noted that a broad TF peak and several sharp peaks resulted. As already mentioned, the trapped field values measured here are still smaller than those of conventional YBCO bulks, but regarding the difference in current density, the porosity and the reduced weight of the foam samples, the results obtained are quite reasonable. Also, most IG-processed bulks are even currently found to exhibit still smaller trapped fields as the melt-textured, melt-growth-processed YBCO bulks [37]. Furthermore, the present foam sample is now already 15 years old and has underwent many experiments as shown in Figs. S1 and S2 in the Supplementary Materials. During this time, the sample was stored in its box without special protection against humidity. This directly demonstrates the chemical stability of the YBCO foam sample.

### 3.2. Current Flow in Foam Samples

A simple estimation of the overall transport current density in the foam sample can be done using the size of the trapped field peak ($B_{\mathrm{trap}}$) with the relation [26]

(1) $$J_{c}R\propto B_{\mathrm{trap},\max}/\mu_{0}$$

where *R* is the radius of the magnetized area [38]. With a trapped field of 250 G and R= 10 mm, we obtain $J_{c}=$ 199 A/cm^2^; and for the maximum trapped field of 400 G, $J_{c}=$ 318 A/cm^2^. In case of the small, sharp peaks as in Figure 4 (Side 3), R= 3 mm and $B_{\mathrm{trap},\max}=$ 150 mT, so $J_{c}=$ 398 A/cm^2^.

Due to the multi-peak trapped field distribution as seen in Figure 3 and Figure 4, this estimation gives the lower boundary for the real critical current density flowing in the sample. This observation points out that the current flow in such porous samples is more complex as in bulk samples, so we need a closer look to it.

Concerning the current flow in porous high-$T_{c}$ materials, we have to be aware that these are polycrystalline materials, so that we have to consider two different contributions to $j_{c}$; the intergranular ($j_{c,\mathrm{inter}}$) and intragranular ($j_{c,\mathrm{intra}}$) currents. The intragranular currents are controlled by the flux pinning and are as high as those of single crystals or bulk samples, whereas the intergranular currents are affected by the grain boundaries between the superconducting grains and by the relative orientations of the anisotropic granules to each other. This describes the case of dense, bulk polycrystalline samples. In porous samples, the overall currents flowing through the entire sample are limited by the porosity and a randomness of the spacial orientation of the foam struts. This mainfests the main drawback for all kinds of porous superconducting materials produced so far [39,40,41,42,43].

**Figure 5:**
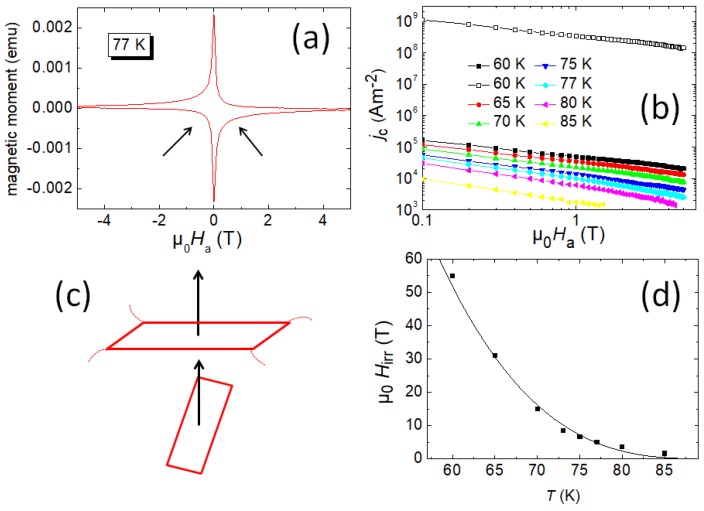
(**a**) Magnetization loop at 77 K, measured on a single foam-strut piece. Arrows point to the asymmetry of the loop. (**b**) $j_{c}$ = intergranular current density, for various temperatures (full symbols). Open symbols show an intragranular current density at 60 K (determined as per the text). (**c**) Schematic sketch of foam-strut orientation to the magnetic field. (Upper) situation in the magnetization measurement, (lower) situation in the real foam sample. (**d**) Irreversibility field, $H_{\mathrm{irr}}$ as function of temperature. Solid line is a fit using relation $H_{\mathrm{irr}}\left(T\right)=H_{0}{\left(1-T/T_{c}\right)}^{n}$ with n= 3.

Figure 5a presents a magnetization loop measured on a foam strut piece at 77 K. The granular character can be seen from the asymmetry of the loop (arrows). This behavior gets more pronounced at elevated temperatures. Figure 5b gives a double log-plot of the critical current density, $j_{c}$, measured on a piece of a foam strut (see the yellow rectangle in Figure 1b. This current density represents an intergranular current density, as it is determined using the Bean model with the dimensions of the entire strut. The $j_{c}\left(H\right)$ curves in this plot look nearly linear, and do not show any so-called fishtail effect [44]. The curves can be fitted using a power law, $H^{-\alpha }$, with α ranging between ∼0.52 (60 K) and 0.8 (85 K). The values of α are simillar to ones obtained on YBCO coated conductors [45], indicating dominant weak collective flux pinning. Bending down of the curves at high *H* and high *T* is due to thermal activation. The open symbols show an intragranular current density (60 K) obtained using the same M(H) data, but the procedure for the determination of the current length scale as described in Reference [38]. This procedure yields an average current-carrying length of ∼6 μm. As result, $j_{c}^{\mathrm{intra}}$ is found to be close to conventional melt-textured or IG-processed bulk samples. Therefore, a further interpretation of the data requires the knowledge about the microstructure of such a foam strut.

It must be noted here that the magnetic field in the SQUID experiments is applied perpendicular to the large surface of the foam strut. Regarding Figure 1b, such a strut is rarely oriented in this configuration in its original location in the foam. The texture of the strut corresponds to its original location in the large foam piece. The seed crystal on the top side introduces the same growth sectors to the foam sample like in a coventional bulk sample. Thus, a foam strut exhibits a crystallographic orientation according to its postion within the 3D structure of the foam. This situation is illustrated in Figure 5c. Breaking out a strut from a foam piece and measuring $j_{c}$ with the magnetic field applied perpendicular to the sample surface means that the field configuration is not ideal like it would be when measuring a sample with its (*a,b*)-plane perpendicular to the magnetic field. Therefore, the $j_{c}$ values of struts can be compared to each other, but not to the current flow within the complete foam sample, where all the struts are in the right position according to the seed crystal. According to the angular dependence of $j_{c}$ as measured by Roas et al. [46], the $j_{c}^{\mathrm{inter}}$-data are lower as in the original configuration within the foam sample. This explains the obvious misfit between our $j_{c}$-data (Figure 5b) and the results of the averaged current density obtained from the TF data (Equation (1)).

There is another important result obtained from the SQUID data. The YBCO foam shows much higher values of the irreversibility field, $H_{\mathrm{irr}}$, as presented in Figure 5d. $H_{\mathrm{irr}}$(*T*) values were determined from the closing of the magnetization loops, and for temperatures, where this was not possible, the $H_{\mathrm{irr}}$ values were determined via the flux pinning force scaling as described in detail in Reference [36]. The solid line in Figure 5d is a fit using the relation $H_{\mathrm{irr}}\left(T\right)=H_{0}{\left(1-T/T_{c}\right)}^{n}$ with n= 3, common for many 123-materials [47]. The fit parameter $H_{0}$ employed for the fit is 130 T. The values of $H_{\mathrm{irr}}$ are clearly much higher than those of other YBCO superconductors, being comparable to those of IG-processed, YBCO bulk samples [48,49]. The $H_{\mathrm{irr}}$ reaches 7 T already at 77 K, which renders the measurement already difficult using the normal laboratory equipment [36]. The irreversibility field is known to be strongly dependent on the pinning strength and on the scale of the current circulation [50,51]. As also texture is achieved in the YBCO foam samples [25], this is very promising for future improvement of the overall critical current densites of such foam samples.

### 3.3. Microstructure

**Figure 6:**
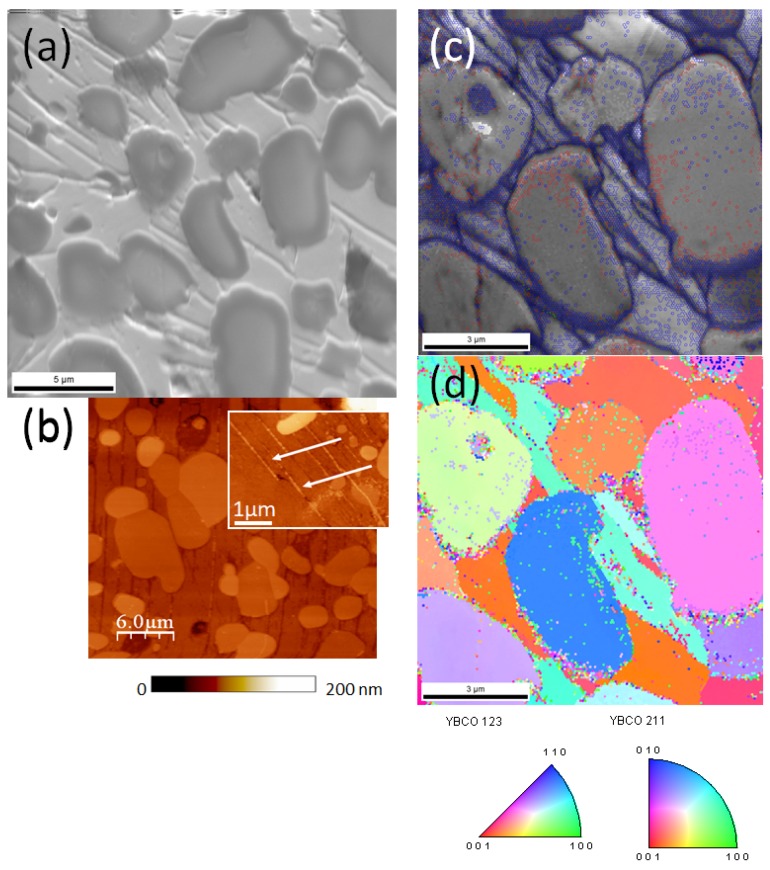
Microstructure of a cross section of a foam strut, investigated by (**a**) SEM, (**b**) atomic force microscopy (AFM) topography, and (**c**,**d**) electron backscatter diffraction (EBSD). (**c**) Image-quality map (resembling a backscattered SEM image) with the EBSD-detected grain boundaries colored in red (1–5° misorientation), green (5–15° misorientation), and blue (15–180° misorientation). (**d**) Orientation map in the [001]-direction. The color code is given in the stereographic triangles below the map. The YBCO matrix shows two dominating orientations, whereas the 211 particles (both large and tiny ones) are randomly oriented.

For achieving high critical current densities in YBCO foam samples, the texture is essential as the grain boundaries in a polycrystalline material will limit the possible current flow [1]. Therefore, it is important to investigate the microstructure of the foam struts as shown in Figure 6. Figure 6a gives a backscattered electron image of a mechanically polished cross section of a foam strut. Here we can see several large 211 grains embedded in the superconducting YBCO matrix. This matrix shows several characteristic stripes, which are due to either grain boundaries or sub-grain boundaries. In Figure 6b, an AFM topography scan of the same sample is presented. The mechanically harder 211 particles are more clearly visible, and at higher magnification (inset), we can see a large amount of very tiny 211 particles located within the stripes (arrows). Figure 6c,d give EBSD orientation mappings of a small section of the strut. Figure 6c is an image quality (IQ) map (resembling a backscattered electron image), together with the EBSD-detected grain boundaries (GBs) drawn in red (1–5° misorientation), green (5–15° misorientation) and blue (15–180° misorientation). The majority of the detected GBs are high-angle GBs, mostly located in the stripes and around the tiny 211 particles. This implies that the current flow through these GBs is somewhat hindered, as a more single-crystalline orientation of the YBCO matrix would allow much higher currents to flow unaltered. On the other hand, the tiny 211 particles are excellent flux pinning sites, so the foam material benefits of their presence, resulting in the improved irreversibility fields as seen in Figure 5.

Figure 6d shows an orientation map in [001]-direction, i.e., perpendicular to the sample surface. The color code is given in the stereographic triangles below the map. The YBCO matrix shows two dominating orientations (±30° off the [001]-direction (red)). In contrast to this, the 211 particles (both large and tiny ones) are randomly oriented. This observation implies that the foam strut shows a texture, but due to the spacial orientation of the strut within the foam sample, the measured orientation cannot be in [001]-direction as in a conventional bulk sample. Therefore, the currents flowing through a strut face the GBs, limiting the maximal currents. Thus, the currents measured by magnetization measurements are clearly lower than those of bulk samples [36]. The improvement of the current densities within the foam struts will be the main challenge for the future development of superconducting foam samples, and a key to this will be an improvement of the texture achieved. For this purpose, the recent developments concerning the generic seed crystals [52], the IG-process [31,37], the pre-treatment of the 211 powders [53,54] and chemical doping to improve texture [55,56] will have to be applied to the fabrication of foam samples. Nevertheless, our first results are promising, so thus we may discuss possible applications of the foam samples to set directions and goals for the coming improvements.

## 4. Applications of Superconducting Foam Samples

The superconducting YBCO foams could see many different applications making use of the efficient cooling process, e.g., for fault current limiters. This was already proposed in the first publications on foams [16,17]. Cooling of superconducting foam structures from room temperature to 77 K proceeds about ten times faster compared to respective bulk materials of the same mass and composition [11]. This can directly be demonstrated when cooling a foam sample in liquid nitrogen, e.g., to test the levitation properties on a magnetic rail consisting of 3 Nd–Fe–B magnets in a row (S–N–S configuration, [57]) as shown in Figures S1 and S2 and in the video sequence in the Supplementary Materials. When operating on such a magnetic rail, the foam sample is cooled so well that it takes ∼5 min to warm up again. Furthermore, the lift height of the foam sample is much higher than that of an YBCO bulk sample due to the reduced weight. Another interesting possibility would be to turn around the situation of the levitation experiment: Using long pieces of foam would create an easily coolable superconducting rail, where magnetic objects can levitate upon. Such a superconducting rail system could be cooled very effectively by any gaseous or liquid coolant being pumped through.

Trapped field magnets can be sources of magnetic fields in places where the standard technology to generate large magnetic fields will not work. Such examples are:

- TF magnets can be applied as static or rotating elements in electric motors and generators (see, e.g., the review in Reference [58]). Here, the reduced weight of the foam samples is very promising. This also applies to magnetic coupling elements in the power line, which is employed to transmit power into a container with different atmospheric conditions. Such lightweight machinery and coupling elements may be useful for space applications.
- TF magnets are portable systems. As long as the system is kept below superconducting transition temperature $T_{c}$, the magnetic field stays in the sample. Using persistent current mode, there is no need for any power supply.
- Combined with pulsed magnetic fields (PMF) [59,60], superconducting samples could be activated as TF magnets when needed, or serve as field receptors only when being cooled.

The points presented here set the possible uses for light-weight TF magnets, but are not yet all application possibilities for superconducting foam samples. Superconducting foams with small open porosity can easily be continuously reinforced, e.g., with resins, to improve their mechanical properties and, thus, to overcome the forces encountered in levitation and quasi-permanent magnet applications [8]. Also an electroplating process to further improve the mechanical strength as done for metallic Al foams [14] could be carried out here. This will avoid the cracking problems of bulk superconductor samples prevailing when trapping large magnetic fields at low temperatures. All these improvements can be applied to the YBCO foam samples, contributing to bring these applications to reality.

Two main applications of superconducting foam samples in space engineering can be envisaged: The foam samples may play an important role for the pinning force docking interfaces (FPDI) discussed in Refs. [18,19]. The supercoducting material required in this application needs to be of large size, i.e., several centimeters in diameter. This size is still out-of-reach for conventional bulk samples, and the weight of such a sample will be considerable, and cracking of the sample may be an important issue. The FPDI system makes use of the stiff field-trapping capability of the superconducting material. One satellite would be equipped with a permanent magnet, and the other one with a superconducting element including a cryocooler. This approach will clearly simplify the docking process between two spacecrafts.

The other space application would be the collection of waste debris in space [20,21,22] using a large magnetic field. The required system could be integrated in a satellite when using a superconducting TF magnet. A cryocooler would keep the superconducting sample at the required temperature to maintain the magnetic field. If not needed anymore, the sample may warm up above $T_{c}$ and the system is demagnetized. To switch on again, a PFM pulse may be employed. In this way, the system is capable to be swtiched on and off on demand.

The large amount of possible applications of foam samples suggest that further development of this sample type is an important task for the future. Combining the latest developments concerning the IG-processing of HTSc materials, the newly developed large seed crystals, the optimization of the sample shape for the given task and improved understanding of the open porous structure via extensive modelling of the 3D structure [61,62] can lead to unique types of superconducting samples to fulfill different demands of applications.

## 5. Conclusions

To conclude, porous high-$T_{c}$ superconductors are very promising materials for applications wherever the sample weight or the cooling efficiency counts. This is given, e.g., in space experiments, for fault current limiters, and trapped field applications in rotating machinery. The achieved trapped field values are still small, but promising and the cooling efficiency can be demonstrated in simple levitation experiments. The future work will require also a deeper understanding of the current flow in such samples, combining already existing modelling approaches of mechanical properties of metallic foams with modelling of the superconducting current flow. Thus, the superconducting foams may be a handy way to generate large magnetic fields in space.

## Supplementary Materials

The following are available online at https://www.mdpi.com/1996-1944/12/6/853/s1, Figure S1: YBCO foam sample being pushed while levitating on a magnetic rail, Figure S2: Superconducting foam levitating above a magnetic rail. (a) Foam sample running at full speed on the rail, and (b) foam sample when being close to warm-up, Video S1: Demonstration of levitating foam sample.
