# Supplementary material for: Superconducting YBCO Foams as Trapped Field Magnets
Source: Materials (Basel). 2019 Mar 13;12(6):853. doi: 10.3390/ma12060853 (PMC6471299; doi:10.3390/ma12060853)
Supplement: Supplementary file 1 [file materials-12-00853-s001.zip › materials-436141-supplementary.pdf]

materials

Supplementary Material

Video sequence of a superconducting YBCO foam, cooled down with liquid nitrogen, operating on a magnetic rail. Sample was field-cooled in a styrofoam container on the magnetic rail. The sample was pushed by hand and, when moving on the rail, one could see nitrogen steaming out of the foam sample, which was deeply cooled due to the open porous structure. Due to the reduced sample weight, the lift height of the foam sample was clearly higher than that of a conventional YBCO bulk sample. At the top of the sample, one can still see remnants of the former seed crystal used during the infiltration growth process.

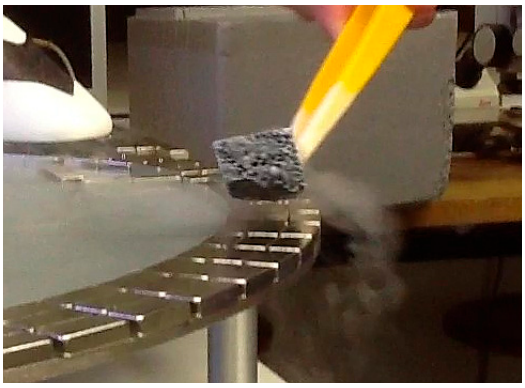

Figure S1. YBCO foam sample being pushed while levitating on a magnetic rail.

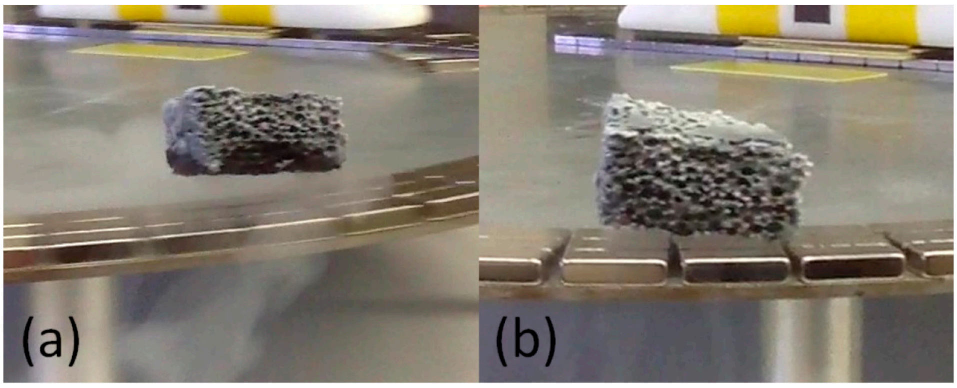

Figure S2. Superconducting foam levitating above a magnetic rail. (**a**) Foam sample running at full speed on the rail, and (**b**) foam sample when being close to warm-up.
